# Combined large cell neuroendocrine carcinoma with giant cell carcinoma of the lungs: a case report

**DOI:** 10.1186/1477-7819-11-205

**Published:** 2013-08-19

**Authors:** Satoshi Hayashi, Masahiro Kitada, Kei Ishibashi, Yoshinari Matsuda, Naoyuki Miyokawa

**Affiliations:** 1 Respiratory Center, Asahikawa Medical University Hospital, Midorigaoka-Higashi 2-1-1-1, Asahikawa, Hokkaido, Japan; 2 Department of Pathology, Asahikawa Medical University Hospital, Midorigaoka-Higashi 2-1-1-1, Asahikawa, Hokkaido, Japan

**Keywords:** Combined large cell neuroendocrine carcinoma, Lung, Giant cell carcinoma

## Abstract

Combined large cell neuroendocrine carcinoma of the lungs (combined LCNEC) with giant cell carcinoma is extremely rare. A 65-year-old man was found to have an abnormal shadow in his left lung field. Computed tomography revealed a solid, round mass measuring 2.8 × 2.2 cm that was located in the left S9. The patient underwent left lower lobectomy and mediastinal lymph node dissection. Histopathological examination revealed an LCNEC, combined with giant cell carcinoma. The patient received by S-1 (TS-1, an oral fluoropyrimidine) chemotherapy, and he has been disease-free for over 8 months. Combined LCNEC with giant cell carcinoma is an extremely rare tumor with high malignant potential, and thus, multidisciplinary therapy and close follow-up are advised.

## Background

Combined large cell neuroendocrine carcinoma (combined LCNEC) is a rare histological type of primary lung carcinoma that has components of adenocarcinoma, squamous cell carcinoma, giant cell carcinoma, and/or spindle cell carcinoma. In 1999, the World Health Organization (WHO) categorized combined LCNEC as a variant of large cell carcinoma (LCC). Combined LCNEC accounts for only 10.6% of all LCNECs. In this context, we report a case of combined LCNEC with giant cell carcinoma that we encountered recently.

## Case presentation

**Figure 1:**
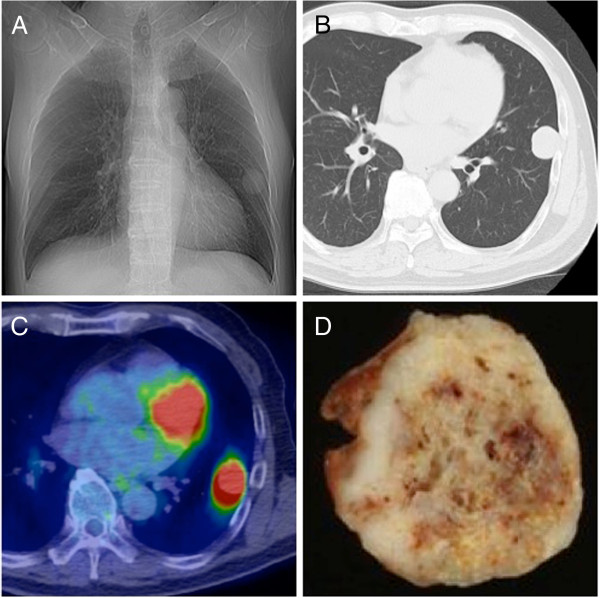
**Imaging findings. (A)** Plain chest radiograph revealed a mass in the left middle lung field. **(B)** Computed tomography (CT) revealed a left lung tumor (28 × 22 mm) with smooth margin. **(C)** Positron-emission tomography revealed fluorodeoxyglucose accumulation in the mass. **(D)** Macroscopic appearance of the resected tumor. The tumor was solid and white with a smooth margin.

**Figure 2:**
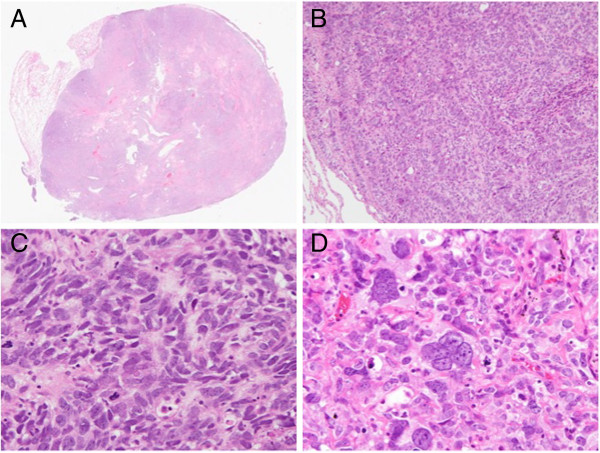
**Histological findings (hematoxylin and eosin).** The tumor was round with a clear margin (**A**, original magnification). The tumor exhibited typical features of large cell neuroendocrine carcinoma (LCNEC) such as palisading (**B**: ×100) and rosette-like structures (**C**: ×400). A few very large, multinucleated, bizarre cells were observed (**D**: ×400).

**Figure 3:**
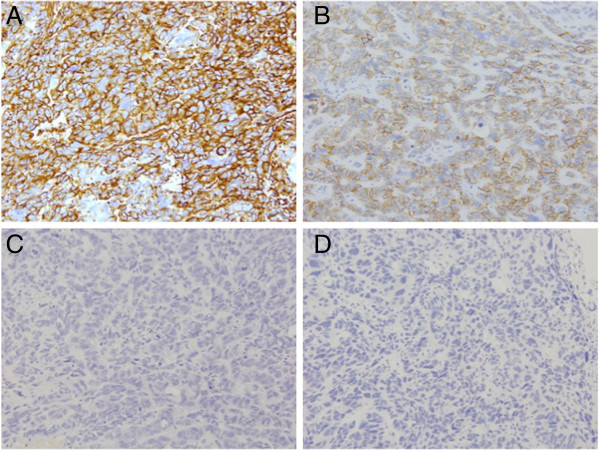
**Immunohistochemical analysis. A**: Vimentin, **B**: NCAM, **C**: Chromogranin A, **D**: Synaptophysin.

A 65-year-old man was found to have an abnormal shadow in his left lung field. A mass was detected in the left middle lung field on a chest radiograph (Figure 1A). Computed tomography (CT) revealed a solid, round mass measuring 2.8 × 2.2 cm located in the left S9 (Figure 1B). No mediastinal lymph node swelling or other organ metastases were observed. On positron emission tomography with ^18^F-fluorodeoxyglucose examination, the maximum standardized uptake value was 12.1, which corresponded to the tumor findings on CT (Figure 1C). Biochemical blood examinations did not reveal abnormalities, and the levels of the tumor markers carcinoembryonic antigen (CEA), squamous cell carcinoma antigen, Cyfra21-1/cytokeratin 19 fragment, and pro-gastrin-releasing peptide were 3.0 ng/ml, 1.9 ng/ml, 2.1 ng/ml, and 30.9 pg/ml, respectively. The patient had received radiation therapy (66 Gy/33 fr) and chemotherapy (super selective intra-arterial chemotherapy, cisplatin 170 mg/body, four courses) for hypopharyngeal squamous cell carcinoma 3 years ago. After the chemotherapy, a complete response was noted. The patient was a smoker with a Brinkman index of 2400. Transbronchial lung biopsy did not facilitate the diagnosis of malignancy. After these examinations, we suspected the tumor to be metastatic or primary lung carcinoma. Surgical biopsy and treatment were selected. The tumor was diagnosed as primary lung carcinoma upon intraoperative diagnosis. Left lower lobectomy and mediastinal lymph node dissection were performed via video-assisted thoracic surgery. The macroscopic specimen was solid and white with a smooth margin (Figure 1D). The actual tumor was 35 × 25 × 22 mm. Histological examination revealed that the tumor was round with a clear margin (Figure 2A). The tumor exhibited typical features of LCNEC such as palisading (Figure 2B) and rosette-like structures (Figure 2C). The tumor cells were large with abundant and foamy cytoplasm. In addition, a few very large, multinucleated, bizarre cells were observed (Figure 2D). Immunohistochemical analysis revealed diffuse and strong staining for vimentin, neural cell adhesion molecule (NCAM), and neuron-specific enolase (Figure 3). The tumor had a keratin profile of AE1/AE3 (+), CK7 (+), and CK5/6 (+), and it was determined to be primary lung cancer (data not shown). The tumor was thus diagnosed as combined LCNEC with giant cell carcinoma, pl2, lymphatic permeation (−), blood vessel invasion (+), and p-T2aN0M0 stage IB. The patient received S-1 (TS-1, an oral fluoropyrimidine) chemotherapy. We have performed close follow-up (every 2 months by CT); no sign of recurrence has been noted for 8 months after surgery.

## Conclusions

According to the WHO classification, combined LCNEC is categorized as LCNEC with components of adenocarcinoma, squamous cell carcinoma, giant cell carcinoma, and/or spindle cell carcinoma [1]. In total, 10.6% of LCNECs are classified as combined LCNEC, and components of combined LCNEC are adenocarcinoma (33.3%), squamous carcinoma (53.3%), and others (13.3%) [2]. Thus, combined LCNEC with giant cell carcinoma is rare.

Preoperative diagnosis of combined LCNEC is very difficult. LCNEC usually appears as a well-defined and lobulated tumor with no air bronchograms or calcification [3], but this is not a specific sign. Therefore, the diagnosis of combined LCNEC is primarily dependent on pathological examination. In our case, CT revealed a solid, round mass. Differential diagnosis also includes primary lung carcinoma, metastatic carcinoma, or benign tumor; thus, histopathological analysis is required for a definitive diagnosis.

Immunohistochemical analysis is important for diagnosing LCNEC. Occasionally, LCNEC resembles the other variants of LCC. The differential diagnosis is dependent on the expression of neuroendocrine markers. With regard to LCNEC, TTF-1, 34βE12 (cytokeratin 1.5.10.14), chromogranin A, synaptophysin, and NCAM, are expressed in 40.9%, 2.2%, 68.5%, 84.2%, and 91.2% of tumors, respectively [4].

There is no standard therapy for combined LCNEC. Combination therapy for LCNEC may now be appropriate in patients with combined LCNEC. Because LCNECs have a poor prognosis, surgery alone is not sufficient. Sakaria and colleagues [5] reported that the response rate of LCNEC to platinum-based neoadjuvant chemotherapy was 68%. In addition, univariate analysis revealed that platinum + etoposide chemotherapy improved the overall survival (OS) of patients with advanced-stage, completely resected LCNEC [5]. In our case, we selected S-1 for adjuvant chemotherapy because the patient previously received cisplatin for hypopharyngeal squamous cell carcinoma, and he did not want to receive aggressive chemotherapy. Platinum-based chemotherapy should be considered for patients who have not previously received platinum-based chemotherapy.

Although LCNEC is categorized as a variant of LCC [1], the biological behavior of LCNEC tumors resemble that of small cell lung carcinoma (SCLC). Battafarano and colleagues [6] demonstrated that, according to histologic subtype, OS at 5 years was 30.2% for patients with LCNECs and 30.3% for patients with combined LCNECs. In view of this finding, LCNEC, combined LCNEC, and SCLC have equivalent prognoses. In patients with stage I LCNEC, the 5-year survival rate was 57.8%, compared with 31.9% for those with stage II LCNEC [2]. Close observation is necessary.

In our case, staining for NCAM, which is located in the cell membrane, was positive. However, staining for synaptophysin and chromogranin A, which are secretory granules was negative. The significance of neuroendocrine (NE) markers remains controversial. NE differentiations may be involved in the prognosis of NE lung cancer. Howe and colleagues [7] reported that the presence of immunohistochemically detected NE differentiation in NSCLC was not of prognostic significance. Conversely, among approximately 116 patients with stage III/IV non-small cell lung carcinoma (NSCLC), the chemotherapeutic response to paclitaxel and cisplatin was significantly better in the NSCLC patients with NE differentiation [8]. They showed that the percentage of NE-positive tumor cells was an independent prognostic factor associated with a favorable outcome. Another report [9] revealed that the number of NE markers was important for predicting the prognosis of LCC. OS was better in LCCs with two or more NE markers [9]. Therefore, we believe that NE markers are potential prognostic factors for lung cancer.

In summary, because combined LCNEC with giant cell carcinoma is an extremely rare tumor with high malignant potential, multidisciplinary therapy and close follow-up are advised. NE markers may have potential as therapeutic and prognostic predictors.

## Consent

Written informed consent was obtained from the patient for publication of this Case report and any accompanying images. A copy of the written consent is available for review by the Editor-in-Chief of this journal.

## Abbreviations

CEA: Carcinoembryonic antigen; CT: Computed tomography; LCC: Large cell carcinoma; LCNEC: Large cell neuroendocrine carcinoma; NCAM: Neural cell adhesion molecule; NE: Neuroendocrine; NSCLC: Non-small cell lung carcinoma, overall survival; SCLC: Small cell lung carcinoma.

## Competing interests

The authors declare that they have no competing interests.

## Authors’ contributions

SH operated this case and analyzed all data. MK, KI, and YM provided assistance for the operation. NM diagnosed the pathology of this case. All authors read and approved the final manuscript.
